# Associations of Depressive Symptoms with Subjective Cognitive Decline in Elderly People—A Cross-Sectional Analysis from the AgeWell.de-Study

**DOI:** 10.3390/jcm12165205

**Published:** 2023-08-10

**Authors:** Isabel Zöllinger, Alexander Bauer, Iris Blotenberg, Christian Brettschneider, Maresa Buchholz, David Czock, Juliane Döhring, Catharina Escales, Thomas Fankhaenel, Thomas Frese, Wolfgang Hoffmann, Hanna Kaduszkiewicz, Hans-Helmut König, Melanie Luppa, Anke Oey, Alexander Pabst, Linda Sanftenberg, Jochen René Thyrian, Julian Weiss, Flora Wendel, Birgitt Wiese, Steffi G. Riedel-Heller, Jochen Gensichen

**Affiliations:** 1 Institute of General Practice and Family Medicine, University Hospital of LMU Munich, 80336 Munich, Germany; linda.sanftenberg@med.uni-muenchen.de (L.S.); julian.weiss@med.uni-muenchen.de (J.W.); flora.kuehne@med.uni-muenchen.de (F.W.); jochen.gensichen@med.uni-muenchen.de (J.G.); 2 Institute of General Practice and Family Medicine, Martin-Luther-University Halle-Wittenberg, 06112 Halle, Germany; alexander.bauer@medizin.uni-halle.de (A.B.); thomas.fankhaenel@medizin.uni-halle.de (T.F.); thomas.frese@uk-halle.de (T.F.); 3 German Center for Neurodegenerative Diseases (DZNE), Site Rostock/Greifswald, 17489 Greifswald, Germany; iris.blotenberg@dzne.de (I.B.); maresa.buchholz@dzne.de (M.B.); wolfgang.hoffmann@uni-greifswald.de (W.H.); rene.thyrian@dzne.de (J.R.T.); 4 Department of Health Economics and Health Services Research, University Medical Center Hamburg-Eppendorf, 20246 Hamburg, Germany; c.brettschneider@uke.de (C.B.); h.koenig@uke.de (H.-H.K.); 5 Department of Clinical Pharmacology and Pharmacoepidemiology, University Hospital Heidelberg, 69120 Heidelberg, Germany; david.czock@med.uni-heidelberg.de; 6 Institute of General Practice, University of Kiel, 24105 Kiel, Germany; j.doehring@allgemeinmedizin.uni-kiel.de (J.D.); escales@allgemeinmedizin.uni-kiel.de (C.E.); kaduszkiewicz@allgemeinmedizin.uni-kiel.de (H.K.); 7 Institute for Community Medicine, University Medicine Greifswald, 17487 Greifswald, Germany; 8 Institute of Social Medicine, Occupational Health and Public Health (ISAP), Medical Faculty, University of Leipzig, 04103 Leipzig, Germany; melanie.luppa@medizin.uni-leipzig.de (M.L.); alexander.pabst@medizin.uni-leipzig.de (A.P.); steffi.riedel-heller@medizin.uni-leipzig.de (S.G.R.-H.); 9 State Health Department of Lower Saxony, 30449 Hannover, Germany; anke.oey@nlga.niedersachsen.de; 10 Work Group Medical Statistics and IT-Infrastructure, Institute for General Practice, Hannover Medical School, 30625 Hannover, Germany; wiese.birgitt@mh-hannover.de

**Keywords:** depression, elderly, subjective cognitive decline, dementia

## Abstract

To develop effective dementia prevention strategies, it is necessary to understand risk factors, associated factors and early signs of dementia. Subjective cognitive decline (SCD) is the earliest form of dementia. The aim of this study is to assess depression as a factor that is significantly associated with SCD. The data of 1030 general practitioner patients from the AgeWell.de-study (60–77 years; CAIDE dementia risk score ≥ 9) were analysed. A descriptive analysis was conducted using validated instruments like the Geriatric depression scale (GDS), Lubben social network scale (LSNS-6) and education classes according to CASMIN (Comparative Analysis of Social Mobility in Industrial Nations). A multivariate regression model with the dependent variable SCD was calculated. Of the 1030 participants, 5.9% had depressive symptoms and 31.3% SCD. The group with depressive symptoms showed significantly higher body-mass-index (*p* = 0.005), lower education class (*p* = 0.022), lower LSNS-6 score (*p* < 0.001), higher sports activity (*p* < 0.001), and more sleeping problems (*p* = 0.026). In the regression model a higher GDS-score [Odds ratio (OR): 1.219 (*p* < 0.001)], more sleeping problems [OR: 1.550 (*p* = 0.017)] and higher education class [middle/high: OR: 1.474/1.875 (*p* = 0.037/0.004)] were significantly associated with SCD. This study identified depressive symptoms, sleeping problems, and higher education classes as factors associated with SCD, which can represent an early form of dementia.

## 1. Introduction

Dementia is a large health and economic burden in our modern society [1]. The economic burden of dementia is due to dementia often restricting the ability to live independently, which is a reason for care dependency of those affected [2]. This constitutes a large burden on the health care system [2]. There was a severe increase in the number dementia cases over the last years and a further increase is predicted [1,3]. Since there is no curative treatment for dementia a preventive strategy is the only option to reduce this progression [4].

Evidence shows that dementia is associated with modifiable risk factors like physical inactivity or smoking, suggesting that there is at least a preventive potential [5]. This preventive potential for a disease that cannot be cured is comparatively large, since up to 30% of the dementia cases can be attributed to modifiable risk factors [6,7].

There are multiple factors increasing the risk of dementia and among these risk factors is depression [8,9,10]. People who have experienced depression have an approximately two times higher risk of developing dementia [11,12]. People with dementia are found to suffer from depression more often than individuals without dementia [13]. Additionally, depression in itself represents an important public health issue [14].

Liew and Wang et al. were recently able to show that depression and a subjective cognitive decline (SCD) are both independent risk factors for the development of dementia [15,16]. They stated an increased risk for the development of dementia for both depressive symptoms and SCD on their own, as well as an even higher risk when both risk factors co-occur [15,16]. Subjective cognitive decline refers to any self-perceived or subjectively experienced worsening of cognitive function in the absence of impaired performance on cognitive tests [17]. A subjective cognitive decline is only noticed by the affected person and not by any other individuals in the personal environment of the affected person [17]. With a prevalence of 10%, subjective cognitive decline is a fairly common symptom in US-American citizens over 45 years of age [18]. It may represent the early symptomatic manifestation of dementia and Alzheimer’s disease [17]. Alzheimer’s disease is a special subtype of dementia and represents the most common form of dementia [17]. It is characterized by a slow progression with a long presymptomatic phase that may take years to decades [17].

On the other hand, depression also has a negative impact on SCD [19]. Often, individuals in studies on SCD report subthreshold symptoms of depression and even psychiatric disorders, i.e., major depression, which may be a reason for SCD [17].

A decrease in cognitive performance can be caused by depression [20]. Specifically, SCD can be caused by depression [21,22]. Therefore, this study aims to evaluate the potential association between depressive symptoms and SCD. This will aid in improving our knowledge of the associated factors of a subjective cognitive decline, which may represent the earliest symptomatic manifestation form of dementia. Therefore, this study opens new research options to develop a future preventive strategy for a serious disease that so far cannot be cured.

## 2. Materials and Methods

### 2.1. Study Design

This study is a cross-sectional observational analysis of data collected as part of the “AgeWell.de—a multi-centric cluster-randomized controlled prevention trial in primary care” baseline data acquisition. The AgeWell.de-Study is a controlled intervention trial to test a multi-facial intervention to reduce cognitive decline in individuals at risk across Germany [5]. The multi-component intervention for the intervention group is carried out over the course of two years and consists of the following components [5]:

▪ Nutritional advice
▪ An enhancement of physical activity
▪ Cognitive training
▪ An optimization of the individuals’ medication
▪ Managing the individuals’ vascular risk factors
▪ Improving the individuals’ social engagement
▪ Interventions for depressive symptoms, bereavement, and grief

For each component of the intervention a personalized schedule will be planned for each participant to optimally aid their adherence and the success of the intervention [5].

The control group received medical treatment by their general practitioner and general health advice as usual [5].

The primary endpoint of the AgeWell.de-Study are changes in the cognitive performance [5]. Secondary endpoints were death, the move to an assisted living facility or a nursing home, activities of daily living, life quality, depressive symptoms, social integration, motivation for a change in behavior, and cost effectiveness [5]. The baseline assessment was conducted from June 2018 to November 2019 at five study sites across Germany [23].

Baseline Assessment

For baseline assessment of the AgeWell.de-Study a home visit for data acquisition was administered by study personnel. This survey was conducted by trained interviewers, using a standardized questionnaire. The individuals were asked questions regarding sociodemographic information, subjective cognitive decline, self-perceived disabilities and disease symptoms, anthropometry, nutrition, physical activity, grief and bereavement, and medication [5]. Questions regarding the primary and secondary endpoints were also addressed during the baseline assessment [5]. Further data regarding the health status, medical diagnoses, lab data, and medication were provided by the general practitioners using a standardized form [5].

### 2.2. Participants

We report on 1030 community-dwelling general practitioner patients between the age of 60 and 77 years from the AgeWell.de-Study [23].

Inclusion criteria of the AgeWell.de-Study: an increased CAIDE dementia risk score (cardiovascular risk factors, aging, and incidence of dementia, cut-off- ≥ 9) [5]. The CAIDE elements included age, education, gender, blood pressure, cholesterol, BMI, and physical activity.

Exclusion criteria of the AgeWell.de-Study: conditions prohibiting a safe participation; severe loss of vision, hearing, or communicative ability; insufficient ability to understand, speak or read German; severe mobility impairment; concurrent participation in another intervention trial; and previously diagnosed dementia or dementia suspected by the general practitioner.

Additionally, individuals with a diagnosis of depression were excluded from the AgeWell.de-Study [5]. For this reason, the appearance of depressive symptoms during the baseline assessment can only be expected on a low level.

### 2.3. Instruments

In this work, the outcomes of the baseline questionnaire of the AgeWell.de-Study are used. Among other parameters, we used the results of the validated Geriatric Depression Scale (GDS) and the Lubben Social Network Scale (LSNS-6). To define SCD, the first step was to determine whether a mild cognitive impairment can be identified:

#### 2.3.1. Mild Cognitive Impairment-Score (MCI-Score)

The MCI-score is positive when the person either has self-experienced memory problems and is concerned about them or other people have noticed that the participant has memory problems. Additionally, the persons score in one of the following standardized cognitive tests must be at least 15 standard deviations below the age, gender, and education adjusted normal value: attention (trail making test A) [24], executive functions (trail making test A/B) [24], learning and memory (CERAD (Consortium to Establish a Registry for Alzheimer’s Disease) wordlist) [25,26,27], language (CERAD verbal fluency test) [26,27,28,29], visuo-constructive praxis (CERAD constructional praxis) [26,27,30], and social cognition (reading the mind in the eyes test—revised version) [31,32]. The person must have any or only minimal impairment in daily activities (≤1 impairment in daily activities in the Barthel-Index) [33] and the persons cannot have dementia diagnosis.

#### 2.3.2. Calculation of the SCD

**Figure 1:**
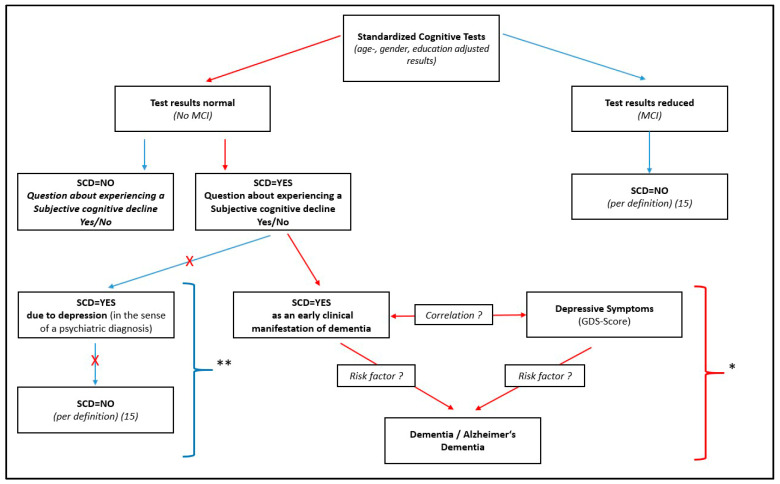
Illustration of the definition of subjective cognitive decline (SCD) and associations with depressive symptoms and dementia. SCD = Subjective cognitive decline; MCI = Mild cognitive impairment. * In this work, correlations of the dementia risk factor depression and the potential risk factor subjective cognitive decline will be analyzed. ** According to the study design, it cannot be determined if a subjective cognitive decline occurs due to depression in the sense of a psychiatric diagnosis. Consequently, this aspect of the SCD is not analyzed in this work.

The SCD was defined according to Jessen et al. [17]. In the first step all persons were included in a “memory-complaints-group”, who affirmed the question “Did you experience any worsening of your memory?”. After that, persons in the memory-complaints-group were excluded who had an objectively measurable impairment in cognitive performance, because normal age-, gender- and education-adjusted performance must be present in standardized cognitive tests to meet the definition of a SCD [17]. This objectively measurable cognitive impairment was judged using the MCI-Score. Additionally, diagnosed depression serves as an exclusion criterion for SCD [17]. This exclusion criterion is already fulfilled by the design of the study, since people with a diagnosis of depression cannot take part in the AgeWell.de-Study [5]. This group of all persons who have self-experienced memory complaints but do not have an objectively measurable cognitive impairment now form the group of persons who have a SCD (SCD group) (See Figure 1).

In order to obtain a more homogeneous group of individuals without a SCD (non-SCD group), all persons with an MCI were also excluded from this group. This means that the non-SCD group is formed by all persons who have no self-experienced memory complaints and no cognitive impairment.

#### 2.3.3. Calculation of the Lubben Social Network Scale

For the calculation of the Lubben social network scale the shortened version of the LSNS-questionnaire with six items (LSNS-6) was used [34]. These questions were selected from the dataset and summed up [34]. This gave a LSNS with a maximum of 30 points, with a higher score indicating more social engagement [34]. Lubben et al. specify that a score <12 indicates that the person is at risk for social isolation [34].

#### 2.3.4. Calculation of the Education Groups According to CASMIN

The education groups were assigned according to the CASMIN classification (Comparative Analysis of Social Mobility in Industrial Nations) [35]. The education groups are split into the three ranks of low, medium, and high [35].

#### 2.3.5. Presence of Sleeping Problems

For the assessment of whether the participant has sleeping problems or not, the participant’s general practitioner was consulted [5]. The general practitioner was asked whether the participant has any kind of sleeping problems diagnosed [5]. The variable sleeping problems is a dichotomous variable, that tells us if the participants sleep is impaired in any form [23]. This variable is based on the clinical expertise of the general practitioners since our study is closely linked to the daily work routine in a doctor’s practice.

#### 2.3.6. Presence of Depressive Symptoms

For the evaluation of depressive symptoms, the validated Geriatric Depression Scale is used [36,37]. A score between 0 and 5 indicates that the person is unaffected from depressive symptoms. For participants with a score ranging from 6 to 10 the GDS indicates that there are light to moderate depressive symptoms. Persons scoring from 11 to 15 are likely to have symptoms of a severe depression. For this analysis a dichotomous approach was chosen. Participants were categorized into the two categories “depressive symptoms” and “no depressive symptoms”. The category “no depressive symptoms” consists of all participants with a GDS-score from 0–5. The GDS-categories “symptoms of a light to moderate depression” and “symptoms of a severe depression” were combined into the category “depressive symptoms” with a GDS-score from 6 to 15. This approach was the most fitting, since having a diagnosis of depression was an exclusion criterion in the AgeWell.de-Study and consequently only a small number of participants with depressive symptoms and just a very small number of participants with severe depressive symptoms was expected.

#### 2.3.7. Selection of Variables for the Descriptive Analysis of Participants with and without Depressive Symptoms

For the descriptive analysis of depressive symptoms, the variables were chosen based on clinical relevance or results from previous similar studies. Previous studies showed significant differences in participants with depressive symptoms compared to participants without depressive symptoms regarding the body-mass-index [38], education level [39,40], social network [41,42,43], physical activity [44,45], and sleeping problems [46].

### 2.4. Trial Registration and Ethical Clearance

The AgeWell.de-Study was registered in the German Clinical Trials Register (DRKS; trial identifier: DRKS00013555) and approved by the responsible ethical board (the Ethics Committee of the Medical Faculty of the University of Leipzig; ethical vote number: 369/17-ek; 12 October 2017) [23]. Participants who participated in the study signed a form to declare their consent to the processing of their data for research purposes as part of the AgeWell.de-Study [5].

### 2.5. Statistical Analyses and Procedure

Metric variables were tested for normal distribution using the Shapiro–Wilk test. If normally distributed they are reported as mean and standard deviation, if not, they are reported as median and interquartile range. Categorial variables are reported as percentages.

It was estimated that the proportion of missing data was low and was missing completely at random. Therefore, the analysis was conducted without imputation with *n* = 1016 and *n* = 1022 participants, correspondingly.

The descriptive analysis is conducted for the whole population and stratified for depressive symptoms and SCD. For group comparisons in the case of categorial variables, the variables were cross-tabulated and statistically assessed using the Participants-chi-squared test. In case of metric variables, the t-test was used for variables with normal distribution and the Mann–Whitney U test was used for variables without normal distribution.

A multivariate logistic regression model was created to evaluate the influence of multiple factors on SCD, which was defined as the dependent variable in the model. Independent variables were selected based on clinical relevance. They were included in the regression model by stepwise selection. Their association with SCD was first analyzed with a univariate regression. Afterwards, all variables with a significant association with SCD were included stepwise in the multivariate regression model and non-significant variables were again excluded stepwise. *p*-values were calculated for the quantitative assessment of these associations.

*p*-values of *p* < 0.05 were considered significant. The statistical analysis was performed utilizing IBM SPSS Statistics.

## 3. Results

### 3.1. Prevalence of Depressive Symptoms and Description of the Differences between Study Participants with and without Depressive Symptoms

Of 1016 participants, 956 (94.1%) presented any symptoms of depression according to their GDS-score (GDS-score ≤ 5). There were 56 (5.5%) participants who presented symptoms of a possible mild to moderate depression (GDS-score = 6–10) and 4 (0.4%) presented symptoms for a possible severe depression (GDS-score = 11–15). This accounts for 60 (5.9%) participants with depressive symptoms according to their GDS-score. The prevalence of depressive symptoms is displayed in Table 1.

After splitting the participants in two groups according to the GDS-score (depressive symptoms at a GDS-score > 5), among the groups with depressive symptoms the median age was 67 years, 55.0% were female, and a corresponding 45.0% were male. Regarding these factors, there was no significant difference compared to the participants without depressive symptoms; however, it could be established that participants with depressive symptoms had a significantly higher body-mass-index than people without depressive symptoms [32.9 kg/m^2^ compared to 30.5 kg/m^2^ (*p* = 0.005)].

Furthermore, there was a significant difference in the distribution of the three education classes according to CASMIN [with depressive symptoms: education high: 10.0%, medium: 55.0%, low: 35.0%; without depressive symptoms: education high: 23.4%, medium: 53.2%, low: 23.4% (*p* = 0.022)]. The proportion of people with medium education was similar between the two groups. However, in the group with depressive symptoms, there was a significantly higher percentage of people with low education and a significantly lower number of people with high education than in the group without depressive symptoms.

Participants were asked if they did at least 30 min of sports a minimum of two times a week. This was more often affirmed by participants who had depressive symptoms [with depressive symptoms: yes: 64.4%, no: 30.5%; without depressive symptoms: yes: 46.8%, no: 52.6% (*p* < 0.001)]. Furthermore, participants with depressive symptoms had a significantly lower LSNS-Score [11.0 compared to 18.0 (*p* < 0.001)] and experienced significantly more sleeping problems [with depressive symptoms: sleeping problems yes: 31.7%, no: 68.3%; without depressive symptoms: sleeping problems yes: 17.9%, no: 81.5% (*p* = 0.026)].

Additionally, a descriptive analysis concerning the variables hypertension, MoCA-Score, daily alcohol consumption and packyears was conducted. However, no significant associations between these variables and depressive symptoms could be found.

The full data of the characteristics and group differences in participants with and without depressive symptoms is displayed in Table 2.

### 3.2. Prevalence of SCD and Description of the Differences between Study Participants with and without SCD

Out of 1022 participants, only 101 (9.9%) participants had an MCI but a majority of 921 (90.1%) participants showed no MCI. Again, 614 of the 1022 participants (60.1%) had reported no self-experienced memory problems and 408 (39.9%) participants had self-experienced memory problems. Of these 408 participants, 320 (31.3%) additionally had no MCI and so form the group of participants that meet the conditions of a subjective cognitive decline (SCD group). The prevalence of SCD is displayed in Table 3.

Looking at the characteristics of the SCD group, among the participants with SCD the median age was 69 years; 52.5% were female and were 47.5% male. However, regarding these factors there was no significant difference compared to the participants without SCD. In contrast, there was a significant difference in the distribution of the three education classes according to CASMIN between the SCD group and non-SCD group, indicating that in the group with SCD the level of education was higher in general. That means that in the SCD group, there was a larger percentage of people with high education and a smaller proportion of people with low education compared to the group without SCD [SCD group: education high: 26.3%, medium: 54.7%, low: 19.1%; non-SCD group: education high: 20.8%, medium: 52.9%, low: 26.3% (*p* = 0.024)].

Furthermore, among the people with SCD, there were more participants who experienced sleeping problems [SCD group: sleeping problems yes: 23.8%, no: 75.3%; non-SCD group: sleeping problems yes: 15.0%, no: 84.7% (*p* = 0.002)]

In addition, a descriptive analysis regarding the variables BMI, Lubben Social Network Scale, two times a week a minimum of 30 min of sports, hypertension, MoCA-Score, daily alcohol consumption and packyears was performed. However, no significant associations between these variables and a subjective cognitive decline could be found. The group differences in participants with and without SCD are presented in Table 2.

### 3.3. Prevalence of Depressive Symptoms and SCD

Among the people with depressive symptoms, there were significantly more participants who experienced a SCD [with depressive symptoms: SCD group: 58.3%, non-SCD group: 41.7%; without depressive symptoms: SCD group: 33.2%, non-SCD group: 66.8%; (*p* < 0.001)].

The same result was shown when comparing the groups with and without SCD according to the presence of depressive symptoms [SCD group: with depressive symptoms: 8.9%, without depressive symptoms: 91.1%; non-SCD group: with depressive symptoms: 3.3%, without depressive symptoms: 96.7% (*p* < 0.001)]. The prevalence of depressive symptoms and SCD are also presented in Table 2.

### 3.4. Multivariate Analysis of Variables That Influence SCD

After analyzing factors that are possibly associated with SCD by examining the differences between the two groups with and without SCD, a multivariate regression model was calculated to evaluate the joint influence these predictors have on SCD. The main aim of the regression model was to evaluate the association of depressive symptoms with SCD.

When comparing the SCD and non-SCD groups, several significant variables could be identified from the multivariate analysis, including sleeping problems, educational attainment according to CASMIN and the GDS-score. Since little social engagement is a known risk factor for SCD, the regression model was also adjusted for the LSNS. It was also decided to adjust for the variables age and sex, despite them not being significant.

The multivariate regression model showed that participants with higher GDS-scores were significantly more likely to have a SCD [OR: 1.219 (*p* < 0.001)]. Additionally, sleeping problems led to an increase in the odds ratio [OR: 1.550 (*p* = 0.017)]. Compared to participants with a low education class, participants with a higher education class were significantly more likely to have a SCD [education group middle: OR: 1.474 (*p* = 0.037); high: OR: 1.875 (*p* = 0.004)]. Controlling for main sociodemographic variables in this regression analyses, a higher age [OR: 1.008 (*p* = 0.584)] and female sex [OR: 0.939 (*p* = 0.670)] also did not significantly change the odds ratio for SCD. Additionally, higher scores in the LSNS-6 showed no significant influence on the likelihood of SCD [OR: 1.023 (*p* = 0.097)].

The full data of the regression model are presented in Table 4.

## 4. Discussion

This analysis showed that depressive symptoms are significantly associated with SCD, confirming previous research [19,22,47]. This result remained significant despite including possible confounders in the multivariate analysis.

### 4.1. SCD-Regression Model

In addition to identifying depressive symptoms as an associated parameter, the regression model also showed that sleeping problems have a significant influence on SCD. Lee et al. and Lin et al. showed that poorer sleep quality has a deteriorative association with SCD [19,48]. Xu et al. also specify depression as a mediating factor for worsening the influence of sleeping problems on SCD [49].

This influence of depressive symptoms on SCD can also be seen in our results of the elevated odds ratio for the GDS-score. This is concordant with other studies which also showed depression to be significantly associated with SCD [19,22,47]. There are also multiple studies showing that depression is a risk factor for the development of dementia [16,48,50,51]. Two studies were able to show more specifically that a previous episode of depression earlier in the person’s life is a risk factor for the later development of dementia and, on the other hand, that a current depressive episode in an elderly person is more likely an early symptom of a prodromal dementia [50,51]. People with depressive symptoms are not only at risk for the development of dementia but also show a poorer current cognitive performance compared to non-depressed participants [52,53].

Furthermore, the multivariate analysis revealed that a higher education level was significantly associated with SCD. This contrasts with other studies which showed that a higher education level decreases the risk of developing dementia [54,55]. Balash et al. showed that education is not significantly associated with SCD [47]. Additionally, Xu et al. showed that fewer years of education do not significantly change the risk of developing dementia [54]. It should be considered that the construct of a SCD is based on symptoms reported by the study participant. Therefore, one explanation for these findings could be that participants with a higher education level recognize self-experienced memory problems more often and thus report them more often.

While not statistically significant, the LSNS-6 was still included in the regression model because other studies have already claimed a deteriorative effect of social isolation on the potential development of dementia [41,43,53]. This could not be further affirmed but the data do not contrast to preceding studies in any way.

### 4.2. Descriptive Analysis of Depressive Symptoms

All participants of the AgeWell.de-Study with at least depressive symptomatology were included in this analysis; however, participants with a diagnosed depression were excluded from the study [5]. Therefore, only a small number of participants with severe depressive symptoms could be expected and the prevalence of depressive symptoms was only 5.9% and lower than in other studies on participants in elderly community dwellings. In the study of Schwarz et al., elderly participants in community dwellings with an age of at least 60 years showed a 27.5% prevalence of depressive symptoms [56]. Stek et al. also had a comparable study population to the community dwelling elderly participants of the AgeWell.de-Study, in participants with an age of at least 85 years they found a 15.4%prevalence of depressive symptoms [57].

Participants with depressive symptoms had a higher body-mass-index. This is concordant with other studies like the meta-analysis of Mannan et al. [38], which postulates an increased risk for depressive people to become obese and vice versa.

The different distribution of the education classes according to CASMIN indicates that there is a higher prevalence of depressive symptoms among people with lower education and a lower prevalence of depressive symptoms in participants with a high education. Studies on this correlation are scarce and often show contrary results to each other. Gan et al. [39] suggest that one explanation could be that the influence of education on depressive symptoms differs regarding the person it affects concerning sex or ethnicity. Gavin et al. [40] showed a decreased risk for white men with a higher education level compared to a lower education level, but an increased risk for white women with a higher education level compared to a lower education level. Among Black, Latino, or Asian men or women no difference could be established [40]. This possible positive effect of higher education on depression could be of interest for future investigations.

Participants with depressive symptoms had a significantly lower LSNS-Score, which indicates them having less social participation. This is in accordance with the studies of Cacioppo et al. [41], Beekman et al. [42], and Domènech-Abella et al. [43], which also found social isolation to be a risk factor for depression.

There are significantly more participants in the group with depressive symptoms who are doing sports, than in the group without depressive symptoms. This contrasts with the study of Schuch and Stubbs [44] and the study of Pearce et al. [45], which both showed that physical activity has a protective potential against depression and can also be used as a treatment for depression.

Participants with depressive symptoms had a significantly higher rate of sleeping problems than participants without. Fang et al. claim that there is a bidirectional relationship between depression and sleeping problems. Sleeping problems can be a symptom of depression, but they can also represent an independent risk factor for the development of depression [46].

### 4.3. Strengths

One strength of the study is that with 1030 cases, we could examine a large number of people that represent an elderly part of the population. Furthermore, for the calculation of the SCD, we used a complex cognitive evaluation with age, gender, and education adjusted scores to evaluate mild cognitive impairment and differentiate between SCD and MCI. This method could be seen as superior compared to a method that only uses scores of unadjusted individual cognitive test results.

### 4.4. Limitations

One limitation of this work is that this is a cross-sectional analysis. Therefore, no causal relationships can be derived from these results.

Another limitation of this work is that only participants who already have an increased risk of developing dementia (increased CAIDE dementia risk score) were included in the AgeWell.de-Study [5]. Additionally, for many variables we were reliant on the truthful self-disclosure of the participant.

For our analysis we especially included all participants who had a SCD. In contrast to another study [47], we did not differentiate between complainers (participants who are worried about their memory problems) and non-complainers (people who are not worried about their memory problems) [47]. Thus, we could not show whether there were possible differences in the prevalence of depressive symptoms between these groups. Since the fact of whether people are concerned about their SCD could possibly indicate their risk of progression to dementia, this could be a relevant factor for a future longitudinal study [47]. However, we conducted a cross-sectional analysis and therefore differentiating between complainers and non-complainers was not of a high relevance for us, since we did not investigate the course of the disease.

Since our research is a cross sectional study, we were not able to show the direction of the identified associations between SCD and depressive symptoms or between SCD and the other associated factors. For example, SCD could be caused by depressive symptoms or vice versa. Despite that, we were still able to show a strong association between these two factors and we could identify multiple other factors associated with SCD.

Due to the general design of the AgeWell.de-Study, only a few participants had severe depressive symptoms, as participants with diagnosed depression were excluded from the study [5]. This did not pose a relevant problem for our analysis as, due to the definition of a SCD, we had to exclude participants with a diagnosed depression anyway. Furthermore, our study aims to investigate SCD and its association with depressive symptoms rather than with a diagnosed depression.

## 5. Conclusions

This study’s descriptive analysis showed that participants with depressive symptoms showed a higher body-mass-index, worse social integration, and a higher rate of sleeping problems. Additionally, participants with depressive symptoms are more likely to be part of a lower education class and less likely to be part of a higher education class than participants without depressive symptoms.

The regression model revealed that depressive symptoms, sleeping problems, and a higher education class are factors that are significantly associated with SCD, which may represent the first symptomatic manifestation for future cognitive decline. The modifiable factors of depressive symptoms and sleeping problems should be of interest for further research to evaluate their effect on the progress of SCD. It could also be of great interest to investigate the benefits of an intervention that improves these factors on the cognitive performance of these individuals.

This study evaluated depressive symptoms, subjective cognitive decline, and the connection between both these parameters in a large elderly population using a complex MCI-Score to judge the participants cognitive performance. Thereby, we were able to further promote our understanding of depressive symptoms in an elderly population, which is an important issue in public health and in subjective cognitive decline, the earliest manifestation of dementia, and a disease that can’t be cured. By identifying demographic characteristics and associated factors we have laid the basis for future research to possibly prevent these major diseases by changing these potential risk factors.

## Figures and Tables

**Table 1:** Prevalence of depressive symptoms according to GDS-Score.

	Absolute Number (*n*)	Relative Number (%)	
No depression (GDS 0–5)	956	94.1%	No Depressive Symptoms according to GDS-Score (GDS 0–5)
Light to moderate depressive symptoms (GDS 6–10)	56	5.5%	Depressive Symptoms according to GDS-Score (GDS 6–15)
Severe depressive symptoms (GDS 11–15)	4	0.4%	
Total	1016	100.0%	

GDS = Geriatric Depression Scale.

**Table 2:** Characteristics of participants with depressive symptoms and subjective cognitive decline.

		Depressive Symptoms According to GDS-Score (*n* = 1016)			Subjective Cognitive Decline (*n* = 921)			All Participants (*n* = 1030)
		Yes (*n* = 60)	No (*n* = 956)	*p*-Value	Yes (*n* = 320)	No (*n* = 601)	*p*-Value	
Age	Median (IQR) [years]	67.00 (10.00)	69.00 (8.00)	0.184	69.00 (9.00)	69.00 (7.00)	0.819	69.00 (8.00)
Sex	Male [*n*]	27 (45.0%)	457 (47.8%)	0.673	152 (47.5%)	293 (48.8%)	0.717	493 (47.9%)
	Female [*n*]	33 (55.0%)	499 (52.2%)		168 (52.5%)	308 (51.2%)		537 (52.1%)
BMI	Median (IQR) [kg/m^2^]	32.85 (6,50)	30.50 (7.70)	0.005 *	30.60 (7.50)	30.50 (7.70)	0.652	30.40 (7.70)
Education group (CASMIN)	Low [*n*]	21 (35.0%)	224 (23.4%)	0.022 *	61 (19.0%)	158 (26.3%)	0.024 *	251 (24.4%)
	Medium [*n*]	33 (55.0%)	508 (53.2%)		175 (54.7%)	318 (52.9%)		546 (53.0%)
	High [*n*]	6 (10.0%)	224 (23.4%)		84 (26.3%)	125 (20.8%)		233 (22.6%)
Lubben social network scale	Median (IQR)	11.00 (7.00)	18.00 (7.00)	<0.001 *	17.00 (7.00)	17.00 (8.00)	0.513	17.00 (8.00)
Two times a week minimum of 30 min of sports	Yes [*n*]	38 (64.4%)	448 (46.9%)	<0.001 *	144 (45.3%)	296 (49.3%)	0.408	489 (47.8%)
	No [*n*]	18 (30.5%)	503 (52.6%)		171 (53.8%)	300 (49.9%)		525 (51.3%)
	Not specified [*n*]	3 (5.1%)	5 (0.5%)		3 (0.9%)	5 (0.8%)		9 (0.9%)
Hypertension	No [*n*]	9 (15.0%)	118 (12.4%)	0.676	46 (14.4%)	66 (11.0%)	0.140	128 (12.4%)
	Yes [*n*]	51 (85.0%)	831 (86.9%)		270 (84.4%)	532 (88.5%)		895 (86.9%)
	Not specified [*n*]	-	7 (0.7%)		4 (1.2%)	3 (0.5%)		7 (0.7%)
MoCA-Score	Median (IQR)	24.00 (7.00)	25.00 (4.00)	0.146	25.00 (4.00)	25.00 (5.00)	0.069	25.00 (4.00)
Sleeping problems	No [*n*]	41 (68.3%)	779 (81.5%)	0.026 *	241 (75.3%)	509 (84.7%)	0.002 *	833 (80.9%)
	Yes [*n*]	19 (31.7%)	171 (17.9%)		76 (23.8%)	90 (15.0%)		191 (18.5%)
	Not specified [*n*]	-	6 (0.6%)		3 (0.9%)	2 (0.3%)		6 (0.6%)
Daily alcohol consumption	Median (IQR) [g/day]	0.00 (9.53)	7.49 (18.26)	0.173	7.49 (17.47)	4.08 (17.47)	0.472	7.49 (17.47)
Packyears	Median (IQR) [py]	7.00 (30.00)	2.00 (26.45)	0.275	0.99 (24.25)	3.60 (27.00)	0.413	2.40 (26.50)
Subjective cognitive decline	No [*n*]	20 (41.7%)	578 (66.8%)	<0.001 *	-			601 (65.3%)
	Yes [*n*]	28 (58.3%)	287 (33.2%)					320 (34.7%)
Depressive symptoms according to GDS-Score	No [*n*]	-			287 (91.1%)	578 (96.7%)	<0.001 *	956 (94.1%)
	Yes [*n*]				28 (8.9%)	20 (3.3%)		60 (5.9%)

IQR = Interquartile range; BMI = Body mass index; Education group (CASMIN) = Education group according to CASMIN; MoCA = Montreal cognitive assessment; GDS = Geriatric depression scale. * = Significant at the 0.05 level.

**Table 3:** Prevalence of subjective cognitive decline (SCD).

	No MCI	MCI	Total
No self-experienced memory problems	601 (58.8%)	13 (1.3%)	614 (60.1%)
Self-experienced memory problems	320 (31.3%) ^#^	88 (8.6%)	408 (39.9%)
Total	921 (90.1%)	101 (9.9%)	1022 (100.0%)

MCI = mild cognitive impairment. ^#^ Participants with SCD.

**Table 4:** Multivariate logistic regression model with subjective cognitive decline as the dependent variable.

	Subjective Cognitive Decline (*n* = 921)	
	OR (95% CI)	*p*-Value
Age	1.008 (0.979–1.038)	0.584
Sex (female)	0.939 (0.701–1.257)	0.670
Sleeping problems *	1.550 (1.080–2.225)	0.017 *
GDS-Score *	1.219 (1.127–1.318)	<0.001 *
Education group CASMIN (low) *	Reference group	0.014 *
Education group CASMIN (medium) *	1.474 (1.024–2–123)	0.037 *
Education group CASMIN (high) *	1.875 (1.224–2.872)	0.004 *
Lubben social network scale	1.023 (0.996–1.050)	0.097

OR = Odds ratio; CI = Confidence interval; GDS = Geriatric depression scale; Education group (CASMIN) = Education group according to CASMIN. * = Significant at the 0.05 level.

## Data Availability

Data are available upon request.

## Abbreviations

CAIDE	Cardiovascular risk factors, aging, and incidence of dementia
CASMIN	Comparative analysis of social mobility in industrial nations
GDS	Geriatric depression scale
SCD	Subjective cognitive decline
MCI	Mild cognitive impairment
MoCA	Montreal cognitive assessment
LSNS-6	Lubben social network scale 6-items

## References

[1] Prince M.J., Wimo A., Guerchet M.M., Ali G.C., Wu Y.T., Prina M. (2015). World Alzheimer Report 2015—The Global Impact of Dementia: An Analysis of Prevalence, Incidence, Cost and Trends.

[2] Anders W., Prince M. (2010). World Alzheimer Report 2010. The Global Economic Impact of Dementia.

[3] Prince M., Bryce R., Albanese E., Wimo A., Ribeiro W., Ferri C.P. (2013). The global prevalence of dementia: A systematic review and metaanalysis. Alzheimer’s Dement. J. Alzheimer’s Assoc..

[4] WHO (2012). Dementia: A Public Health Priority.

[5] Zülke A., Luck T., Pabst A., Hoffmann W., Thyrian J.R., Gensichen J., Kaduszkiewicz H., König H.H., Haefeli W.E., Czock D. (2019). AgeWell.de—Study protocol of a pragmatic multi-center cluster-randomized controlled prevention trial against cognitive decline in older primary care patients. BMC Geriatr..

[6] Luck T., Riedel-Heller S.G. (2016). Prävention von Alzheimer-Demenz in Deutschland: Eine Hochrechnung des möglichen Potenzials der Reduktion ausgewählter Risikofaktoren [Prevention of Alzheimer’s dementia in Germany: A projection of the possible potential of reducing selected risk factors]. Der Nervenarzt.

[7] Norton S., Matthews F.E., Barnes D.E., Yaffe K., Brayne C. (2014). Potential for primary prevention of Alzheimer’s disease: An analysis of population-based data. Lancet Neurol..

[8] Livingston G., Huntley J., Sommerlad A., Ames D., Ballard C., Banerjee S., Brayne C., Burns A., Cohen-Mansfield J., Cooper C. (2020). Dementia prevention, intervention, and care: 2020 report of the Lancet Commission. Lancet.

[9] Diniz B.S., Butters M.A., Albert S.M., Dew M.A., Reynolds C.F. (2013). Late-life depression and risk of vascular dementia and Alzheimer’s disease: Systematic review and meta-analysis of community-based cohort studies. Br. J. Psychiatry J. Ment. Sci..

[10] da Silva J., Gonçalves-Pereira M., Xavier M., Mukaetova-Ladinska E.B. (2013). Affective disorders and risk of developing dementia: Systematic review. Br. J. Psychiatry J. Ment. Sci..

[11] Barnes D.E., Yaffe K. (2011). The projected effect of risk factor reduction on Alzheimer’s disease prevalence. Lancet Neurol..

[12] Jorm A.F. (2000). Is depression a risk factor for dementia or cognitive decline?. A review. Gerontology.

[13] Forsell Y., Winblad B. (1998). Major depression in a population of demented and nondemented older people: Prevalence and correlates. J. Am. Geriatr. Soc..

[14] Moussavi S., Chatterji S., Verdes E., Tandon A., Patel V., Ustun B. (2007). Depression, chronic diseases, and decrements in health: Results from the World Health Surveys. Lancet.

[15] Liew T.M. (2019). Depression, subjective cognitive decline, and the risk of neurocognitive disorders. Alzheimer’s Res. Ther..

[16] Wang S.M., Han K.D., Kim N.Y., Um Y.H., Kang D.W., Na H.R., Lee C.U., Lim H.K. (2021). Late-life depression, subjective cognitive decline, and their additive risk in incidence of dementia: A nationwide longitudinal study. PLoS ONE.

[17] Jessen F., Amariglio R.E., van Boxtel M., Breteler M., Ceccaldi M., Chételat G., Dubois B., Dufouil C., Ellis K.A., van der Flier W.M. (2014). Subjective Cognitive Decline Initiative (SCD-I) Working Group (2014). A conceptual framework for research on subjective cognitive decline in preclinical Alzheimer’s disease. Alzheimer’s Dement. J. Alzheimer’s Assoc..

[18] Schliep K.C., Barbeau W.A., Lynch K.E., Sorweid M.K., Varner M.W., Foster N.L., Qeadan F. (2022). Overall and sex-specific risk factors for subjective cognitive decline: Findings from the 2015-2018 Behavioral Risk Factor Surveillance System Survey. Biol. Sex Differ..

[19] Lin L.H., Wang S.B., Xu W.Q., Hu Q., Zhang P., Ke Y.F., Huang J.H., Ding K.R., Li X.L., Hou C.L. (2022). Subjective cognitive decline symptoms and its association with socio-demographic characteristics and common chronic diseases in the southern Chinese older adults. BMC Public Health.

[20] O’Brien J.T., Lloyd A., McKeith I., Gholkar A., Ferrier N. (2004). A longitudinal study of hippocampal volume, cortisol levels, and cognition in older depressed subjects. Am. J. Psychiatry.

[21] Burmester B., Leathem J., Merrick P. (2016). Subjective Cognitive Complaints and Objective Cognitive Function in Aging: A Systematic Review and Meta-Analysis of Recent Cross-Sectional Findings. Neuropsychol. Rev..

[22] Reid L.M., Maclullich A.M. (2006). Subjective memory complaints and cognitive impairment in older people. Dement. Geriatr. Cogn. Disord..

[23] Röhr S., Zülke A., Luppa M., Brettschneider C., Weißenborn M., Kühne F., Zöllinger I., Samos F.Z., Bauer A., Döhring J. (2021). Recruitment and Baseline Characteristics of Participants in the AgeWell.de Study-A Pragmatic Cluster-Randomized Controlled Lifestyle Trial against Cognitive Decline. Int. J. Environ. Res. Public Health.

[24] Reitan R.M. (1992). Trail Making Test: Manual for Administration and Scoring.

[25] Satzger W., Hampel H., Padberg F., Bürger K., Nolde T., Ingrassia G., Engel R.R. (2001). Zur praktischen Anwendung der CERAD-Testbatterie als neuropsychologisches Demenzscreening [Practical application of the CERAD test battery as a neuropsychological dementia screening test]. Der Nervenarzt.

[26] Morris J.C., Mohs R.C., Rogers H., Fillenbaum G., Heyman A. (1988). Consortium to establish a registry for Alzheimer’s disease (CERAD) clinical and neuropsychological assessment of Alzheimer’s disease. Psychopharmacol. Bull..

[27] Morris J.C., Heyman A., Mohs R.C., Hughes J.P., van Belle G., Fillenbaum G., Mellits E.D., Clark C. (1989). The Consortium to Establish a Registry for Alzheimer’s Disease (CERAD). Part I. Clinical and neuropsychological assessment of Alzheimer’s disease. Neurology.

[28] Isaacs B., Kennie A.T. (1973). The set test as an aid to the detection of dementia in old people. Br. J. Psychiatry J. Ment. Sci..

[29] Tombaugh T.N., Kozak J., Rees L. (1999). Normative data stratified by age and education for two measures of verbal fluency: FAS and animal naming. Arch. Clin. Neuropsychol. Off. J. Natl. Acad. Neuropsychol..

[30] Rosen W.G., Mohs R.C., Davis K.L. (1984). A new rating scale for Alzheimer’s disease. Am. J. Psychiatry.

[31] Baron-Cohen S., Wheelwright S., Hill J., Raste Y., Plumb I. (2001). The “Reading the Mind in the Eyes” Test revised version: A study with normal adults, and adults with Asperger syndrome or high-functioning autism. J. Child Psychol. Psychiatry Allied Discip..

[32] Bölte S. (2005). Reading Mind in the Eyes Test für Erwachsene (dt. Fassung) von S. Baron-Cohen.

[33] Mahony F.I., Barthel D.W. (1965). Functional evaluation, the barthel index; A simple index of independence useful in scoring improvement in the rehabilitation of the chronically ill. Md. State Med. J..

[34] Lubben J., Blozik E., Gillmann G., Iliffe S., von Renteln Kruse W., Beck J.C., Stuck A.E. (2006). Performance of an abbreviated version of the Lubben Social Network Scale among three European community-dwelling older adult populations. Gerontologist.

[35] König W., Lüttinger P., Müller W. (1988). A Comparative Analysis of the Development and Structure of Educational Systems: Methodological Foundations and the Construction of a Comparative Educational Scale.

[36] Sheikh J.I., Yesavage J.A. (1986). Geriatric Depression Scale (GDS): Recent evidence and development of a shorter version. Clin. Gerontol. J. Aging Ment. Health.

[37] Gauggel S., Birkner B. (1999). Validität und Reliabilität einer deutschen Version der Geriatrischen Depressionsskala (GDS). Z. Klin. Psychol..

[38] Mannan M., Mamun A., Doi S., Clavarino A. (2016). Prospective Associations between Depression and Obesity for Adolescent Males and Females—A Systematic Review and Meta-Analysis of Longitudinal Studies. PLoS ONE.

[39] Gan Z., Li Y., Xie D., Shao C., Yang F., Shen Y., Zhang N., Zhang G., Tian T., Yin A. (2012). The impact of educational status on the clinical features of major depressive disorder among chinese women. J. Affect. Disord..

[40] Gavin A.R., Walton E., Chae D.H., Alegria M., Jackson J.S., Takeuchi D. (2010). The associations between socio-economic status and major depressive disorder among blacks, latinos, asians and non-hispanic whites: Findings from the Collaborative Psychiatric Epidemiology Studies. Psychol. Med..

[41] Cacioppo J.T., Hughes M.E., Waite L.J., Hawkley L.C., Thisted R.A. (2006). Loneliness as a specific risk factor for depressive symptoms: Cross-sectional and longitudinal analyses. Psychol. Aging.

[42] Beekman A.T., Deeg D.J., Geerlings S.W., Schoevers R.A., Smit J.H., van Tilburg W. (2001). Emergence and persistence of late life depression: A 3-year follow-up of the Longitudinal Aging Study Amsterdam. J. Affect. Disord..

[43] Domènech-Abella J., Lara E., Rubio-Valera M., Olaya B., Moneta M.V., Rico-Uribe L.A., Ayuso-Mateos J.L., Mundó J., Haro J.M. (2017). Loneliness and depression in the elderly: The role of social network. Soc. Psychiatry Psychiatr. Epidemiol..

[44] Schuch F.B., Stubbs B. (2019). The Role of Exercise in Preventing and Treating Depression. Curr. Sports Med. Rep..

[45] Pearce M., Garcia L., Abbas A., Strain T., Schuch F.B., Golubic R., Kelly P., Khan S., Utukuri M., Laird Y. (2022). Association Between Physical Activity and Risk of Depression: A Systematic Review and Meta-analysis. JAMA Psychiatry.

[46] Fang H., Tu S., Sheng J., Shao A. (2019). Depression in sleep disturbance: A review on a bidirectional relationship, mechanisms and treatment. J. Cell. Mol. Med..

[47] Balash Y., Mordechovich M., Shabtai H., Giladi N., Gurevich T., Korczyn A.D. (2013). Subjective memory complaints in elders: Depression, anxiety, or cognitive decline?. Acta Neurol. Scand..

[48] Lee S.H., Kang Y., Cho S.J. (2017). Subjective cognitive decline in patients with migraine and its relationship with depression, anxiety, and sleep quality. J. Headache Pain.

[49] Xu W.Q., Lin L.H., Ding K.R., Ke Y.F., Huang J.H., Hou C.L., Jia F.J., Wang S.B. (2021). The role of depression and anxiety in the relationship between poor sleep quality and subjective cognitive decline in Chinese elderly: Exploring parallel, serial, and moderated mediation. J. Affect. Disord..

[50] Gutzmann H., Qazi A. (2015). Depression associated with dementia. Z. Gerontol. Geriatr..

[51] Kessing L.V. (2012). Depression and the risk for dementia. Curr. Opin. Psychiatry.

[52] Neuvonen E., Lehtisalo J., Ngandu T., Levälahti E., Antikainen R., Hänninen T., Laatikainen T., Lindström J., Paajanen T., Soininen H. (2022). Associations of Depressive Symptoms and Cognition in the FINGER Trial: A Secondary Analysis of a Randomised Clinical Trial. J. Clin. Med..

[53] Tzang R.F., Yang A.C., Yeh H.L., Liu M.E., Tsai S.J. (2015). Association of depression and loneliness with specific cognitive performance in non-demented elderly males. Med. Sci. Monit. Int. Med. J. Exp. Clin. Res..

[54] Xu W., Tan L., Wang H.F., Tan M.S., Tan L., Li J.Q., Zhao Q.F., Yu J.T. (2016). Education and Risk of Dementia: Dose-Response Meta-Analysis of Prospective Cohort Studies. Mol. Neurobiol..

[55] Lövdén M., Fratiglioni L., Glymour M.M., Lindenberger U., Tucker-Drob E.M. (2020). Education and Cognitive Functioning Across the Life Span. Psychol. Sci. Public Interest A J. Am. Psychol. Soc..

[56] Schwarz R., Gunzelmann T., Hinz A., Brähler E. (2001). Angst und Depressivität in der über 60-jährigen Allgemeinbevölkerung [Anxiety and depression in the general population over 60 years old]. Dtsch. Med. Wochenschr..

[57] Stek M.L., Gussekloo J., Beekman A.T., van Tilburg W., Westendorp R.G. (2004). Prevalence, correlates and recognition of depression in the oldest old: The Leiden 85-plus study. J. Affect. Disord..

